# Digoxin Revisited: An Updated Review in the Light of Recent Trials

**DOI:** 10.14740/cr2287

**Published:** 2026-10-24

**Authors:** Muhammad Saad, Muhammad Abdullah, Ankit Yelamarthy, Krystal John, Abbas Hayat Mallick, Behram Khan, Hassan Tahir, Rajan Krishnamani

**Affiliations:** a Department of Internal Medicine, The Jewish Hospital of Cincinnati, Cincinnati, OH, USA; b Carl and Edyth Lindner Research Center, The Christ Hospital, Cincinnati, OH, USA; c Department of Internal Medicine, FMH College of Medicine and Dentistry, Lahore, Punjab, Pakistan; d Department of Geriatric Medicine, NYU Langone Health, New York, NY, USA; e Department of Cardiology, Rochester Regional Health, Rochester, NY, USA; f Mercy Heart Institute, Cincinnati, OH, USA

**Keywords:** Digoxin, Rheumatic heart disease, Heart failure, Public health

## Abstract

Isolated from *Digitalis* species following William Withering’s 1785 work on foxglove, digoxin inhibits myocardial Na^+^/K^+^-ATPase, raising intracellular calcium and vagal tone to produce inotropic, rate-controlling, and neurohormonal-deactivating effects. The foundational DIG trial (1997) showed no mortality benefit but reduced heart failure hospitalizations, prompting a progressive guideline downgrade. Three decades later, DIGIT-HF (2025, digitoxin) and DECISION (2026, low dose digoxin) retested cardiac glycosides atop modern four pillar guideline-directed medical therapy (GDMT). DIGIT-HF significantly reduced the composite of death or worsening heart failure (hazard ratio (HR), 0.82), while DECISION showed a numerical but nonsignificant benefit. A pooled meta-analysis of DIG, DIGIT-HF, and DECISION (9,013 patients) confirmed a consistent 15% reduction in worsening heart failure events across three decades of changing background therapy, without a significant mortality effect. The Dig-RHD trial addressed an unstudied population: symptomatic rheumatic heart disease (RHD), a chronic valvular heart disease that results from permanent damage to cardiac valves due to group A streptococcal infection. RHD affects roughly 40 million people and causes approximately 300,000 deaths annually, disproportionately in low- and middle-income countries. In 1,769 patients randomized across 12 Indian centers, digoxin significantly reduced the composite of death or new-onset/worsening heart failure (31.4% vs. 35.5%; HR, 0.82; 95% confidence interval (CI), 0.70 to 0.97; P = 0.02), with greater benefit in atrial fibrillation, women, and patients with low body mass index (BMI), and a number needed to treat of approximately 14 over 2 years. Digoxin is not a mortality-reducing drug, but pooled contemporary evidence establishes it as a reproducible, low-cost tool for reducing heart failure events, now supported for the first time by high-quality randomized data in RHD, a population with substantial global burden and no prior evidence-based medical therapy. These findings warrant reconsideration of digoxin’s guideline status and development of RHD-specific recommendations.

## Introduction: History and Discovery of Digoxin

Foxglove-derived compounds have been used therapeutically for centuries, with written references dating back to 1250. However, their scientific legitimacy was established by William Withering, an English physician and botanist, whose 1785 monograph systematically documented the effects of digitalis in the treatment of dropsy, now recognized as edema secondary to congestive heart failure. Although Withering regarded the drug primarily as a diuretic, his careful clinical observations laid the foundation for modern pharmacology, even though its direct cardiac mechanism remained unknown at the time [1, 2].

Following Withering’s publication, digitalis preparations were widely adopted, often with inconsistent dosing. Advances in chemistry during the 19th and 20th centuries led to the isolation of active cardiac glycosides, including digoxin and digitoxin, from *Digitalis purpurea* and the more potent *Digitalis lanata* [2, 3]. These developments enabled standardized formulations and more predictable pharmacokinetics. For much of the 20th century, digoxin remained a cornerstone in the management of congestive heart failure and atrial fibrillation. However, its role progressively narrowed with the emergence of therapies demonstrating clearer mortality benefits and increasing recognition of its narrow therapeutic index [2].

Despite being displaced from first-line therapy in many settings, digoxin has recently re-emerged in a distinct clinical context: heart failure secondary to rheumatic heart disease (RHD). RHD affects an estimated 40 million people worldwide and accounts for approximately 320,000 deaths annually, disproportionately burdening low- and middle-income countries. RHD is a chronic valvular heart disease that results from permanent cardiac valve damage due to acute rheumatic fever (ARF). ARF is an abnormal autoimmune response to group A streptococcal (*Streptococcus pyogenes*) infection, usually pharyngitis, that occurs often in childhood and adolescence [4]. A contemporary randomized controlled trial (RCT) has demonstrated that digoxin reduces the composite risk of death or worsening heart failure in patients with symptomatic RHD, providing high-quality evidence for a therapy long used empirically [5].

This review examines the historical evolution of digoxin, its molecular mechanisms and pharmacologic properties, and its shifting clinical role, with particular emphasis on its emerging evidence-based application in RHD.

## Pharmacology and Mechanism of Action

Digoxin’s principal molecular target is the myocardial sodium-potassium adenosine triphosphatase (Na^+^/K^+^-ATPase) pump (Fig. 1). By reversibly inhibiting this enzyme, digoxin partially dissipates the transmembrane sodium gradient that the cell would normally maintain. The resulting rise in intracellular sodium reduces the ability of the sodium-calcium exchanger (NCX1) to extrude calcium from the myocyte, since that exchanger depends on the sodium gradient to do its work. The net effect is an accumulation of cytosolic calcium, which enhances excitation-contraction coupling and increases the force and velocity of myocardial contraction, the drug’s classic positive inotropic effect [2]. Recent structural and functional work has refined this picture, showing that digoxin’s inotropic action depends specifically on a sodium-dependent allosteric inactivation mechanism of NCX1 itself, rather than simply on bulk changes in the sodium gradient [6].

**Figure 1:**
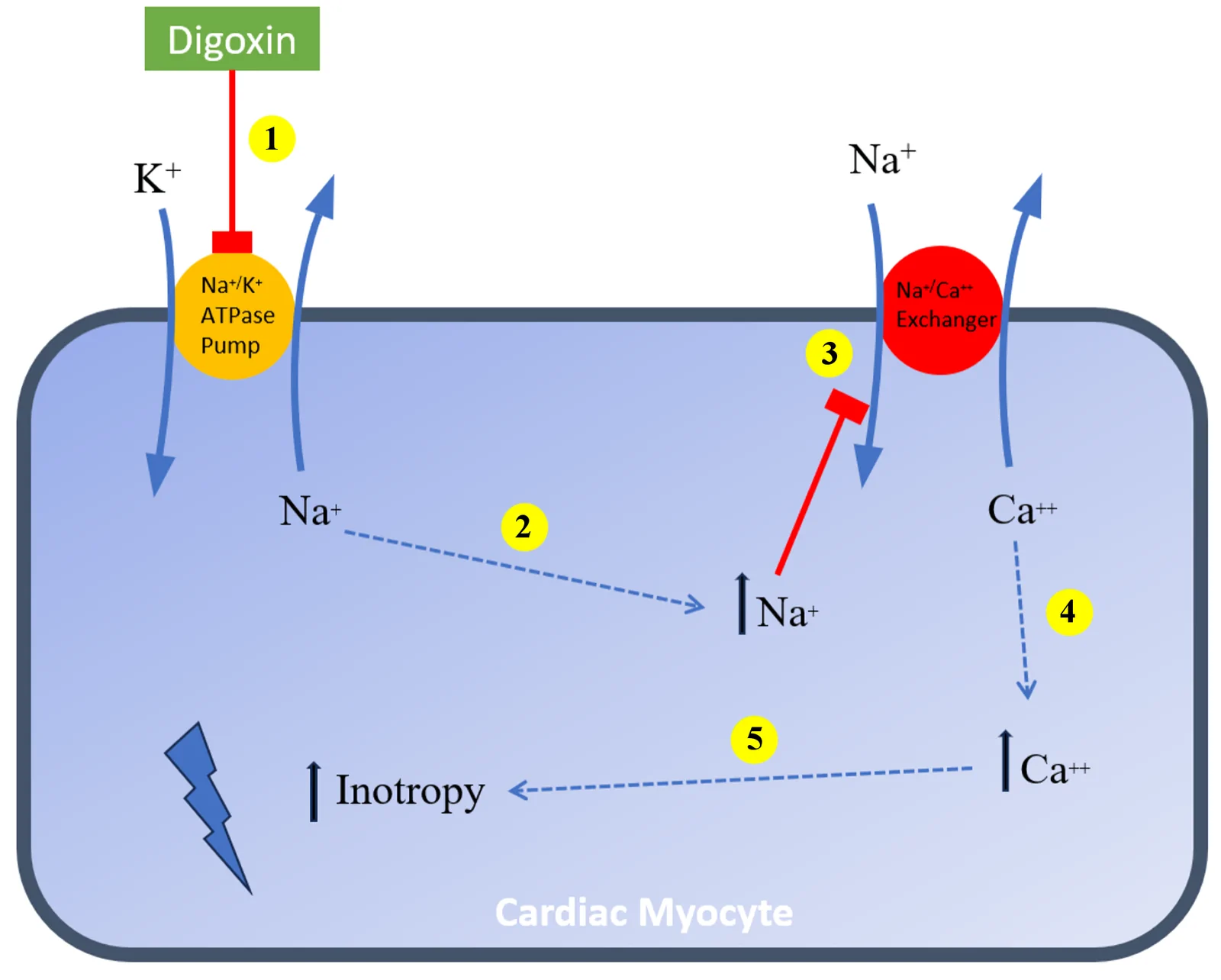
Cellular mechanism of digoxin’s positive inotropic effect. Digoxin inhibits the sarcolemmal Na^+^/K^+^-ATPase pump (1), causing intracellular Na^+^ concentration to rise (2). This increase in intracellular Na^+^ reduces the ability of the Na^+^/Ca^2+^ exchanger (NCX1) to extrude calcium, both by diminishing the transmembrane Na^+^ gradient the exchanger depends on and by triggering allosteric, sodium-dependent inactivation of NCX1 itself (3), reducing Ca^2+^ efflux from the cell. This leads to increased intracellular Ca^2+^ concentration (4), which enhances myocardial contractility (positive inotropy) (5).

Independent of its inotropic action, digoxin exerts a vagomimetic (parasympathomimetic) effect that is central to its modern clinical use. Digoxin inhibits Na^+^/K^+^-ATPase in vagal afferent fibers, which sensitizes cardiac baroreceptors and increases afferent inhibitory signaling to the central nervous system; the resulting increase in vagal tone slows conduction through the atrioventricular (AV) node and prolongs its refractory period. Clinically, this negative dromotropic and negative chronotropic effect is what makes digoxin useful for ventricular rate control in atrial fibrillation and flutter, and it is this indication, rather than heart failure, that carries the strongest current evidence supporting digoxin’s use. Direct depressant effects on the atria and AV node have also been demonstrated but generally require concentrations above the therapeutic range seen clinically [2, 6].

Beyond its direct cardiac electrophysiological effects, digoxin exerts a broader neurohormonal deactivating effect. Vagal afferent sensitization reduces central sympathetic outflow, while inhibition of Na^+^/K^+^-ATPase in the renal tubules reduces sodium reabsorption; the resulting increase in distal sodium delivery suppresses renin secretion. Together, these actions dampen activation of both the sympathetic nervous system and the renin-angiotensin system. Some contemporary work has suggested that in heart failure, these neurohormonal effects, rather than the positive inotropic action for which digoxin was historically valued, may account for much of its clinical benefit, since digoxin has been shown to reduce heart-failure hospitalizations without a demonstrated effect on overall mortality [2].

Digoxin’s clinical usefulness is constrained by a narrow therapeutic index, with substantial overlap between concentrations that are effective and those that are toxic. Guidance on the precise target range has shifted over time: while a serum concentration range of roughly 0.8–2.0 ng/mL was long cited as therapeutic, accumulating evidence associating higher concentrations with increased morbidity and mortality has pushed contemporary recommendations toward lower targets, with some experts and reviews now advising levels closer to 0.5–1.0 ng/mL, particularly in heart failure. Toxicity risk rises appreciably above 2.0 ng/mL, but it is not strictly confined to elevated levels; case reports have documented clinically significant toxicity, including bradycardia and hyperkalemia, occurring at concentrations within the nominally therapeutic range, underscoring that clinical assessment cannot be replaced by a single lab value [7].

Because roughly 70% of digoxin is renally excreted, renal function is a key determinant of dosing and accumulation risk, and levels can rise sharply in patients with declining glomerular filtration. Dosing adjustments are typically guided by renal clearance, ideal body weight (lean mass), age, and concurrent interacting medications (such as P-glycoprotein inhibitors) [8, 9]. Serum concentrations should be drawn at least 6–8 h after the last dose (and ideally just before the next scheduled dose) to avoid capturing the drug during its distribution phase, with levels typically first checked around 7–10 days after initiation, once steady state has been reached. Electrolyte disturbances, particularly hypokalemia, hypomagnesemia, and hypercalcemia, increase myocardial sensitivity to digoxin and can precipitate toxicity even at nominally therapeutic concentrations, so serum electrolytes and renal function warrant periodic reassessment alongside digoxin levels themselves. Numerous drug interactions (notably with agents affecting P-glycoprotein-mediated transport) can also raise digoxin concentrations, making medication reconciliation an important part of ongoing monitoring, especially in older patients who are disproportionately likely to be on multiple interacting drugs.

## The Foundational Evidence: The DIG Trial and the Guideline Era

The evidence for digoxin rests on a remarkably small number of RCTs, making it a unique and underexplored drug—a paradox for one of the oldest agents in cardiovascular medicine [10]. This long-forgotten drug has re-entered discussion thanks to new RCTs highlighting its potential benefit. The foundation for this line of inquiry was laid by the DIG trial (1997), which compared digoxin to placebo on top of angiotensin-converting enzyme (ACE) inhibitors and diuretics. This multicenter, double-blind study enrolled 6,800 patients with heart failure and a left ventricular ejection fraction of 45% or less, all in sinus rhythm. Over a mean follow-up of 37 months, digoxin showed no significant effect on all-cause mortality (34.8% vs. 35.1%; relative risk (RR) 0.99; 95% confidence interval (CI), 0.91–1.07) [2]. It did, however, significantly reduce hospitalizations for worsening heart failure (26.8% vs. 34.7%; RR 0.72; 95% CI, 0.66–0.79; P < 0.001) and showed a trend toward lower heart-failure-specific mortality (RR 0.88; 95% CI, 0.77–1.01; P = 0.06) [11].

Perhaps the most important findings from the DIG trial came from subsequent *post hoc* analyses, which revealed a concentration-dependent relationship between serum digoxin concentration (SDC) and outcomes. Rathore et al (2003) analyzed men with left ventricular ejection fraction (LVEF) ≤ 45% and found that patients with SDC of 0.5–0.8 ng/mL had a 6.3% lower absolute mortality rate compared with placebo, whereas those with SDC ≥ 1.2 ng/mL had an 11.8% higher absolute mortality rate [12]. A comprehensive *post hoc* analysis by Ahmed et al (2006), which included all DIG trial participants with measured SDCs regardless of sex or ejection fraction, confirmed that SDC of 0.5–0.9 ng/mL was associated with reduced mortality (adjusted HR 0.77; 95% CI, 0.67–0.89) and reduced heart failure hospitalizations (adjusted HR 0.62; 95% CI, 0.54–0.72). At SDC ≥ 1.0 ng/mL, heart failure hospitalizations were still reduced (adjusted HR 0.68; 95% CI, 0.59–0.79), but there was no effect on mortality [13]. These findings reshaped clinical practice, shifting the therapeutic target from the previously accepted range of 0.5–2.0 ng/mL down to 0.5–0.9 ng/mL, and laid the groundwork for dosing strategies and trial designs that followed [10].

In the post-DIG era, digoxin was considered standard therapy for heart failure. In the 2005 American College of Cardiology (ACC)/American Heart Association (AHA) heart failure guidelines, it carried a class I recommendation. In the 2009 ACC/AHA focused update, the writing committee explicitly downgraded digoxin from class I to class IIa, level of evidence B, stating that “in terms of safety and efficacy, digitalis does not compare favorably with such agents as the aldosterone blockers” and that “if digitalis were a new drug with clinical trials showing a very narrow risk/benefit ratio and no mortality benefit, it would clearly not be considered for a class I recommendation” [14]. This class IIa status “can be beneficial to decrease hospitalizations for HF” was retained through the 2013 American College of Cardiology Foundation (ACCF)/AHA guidelines [15]. This period was notable because beta-blockers and mineralocorticoid receptor antagonists (MRAs) became established as primary therapies, while digoxin came to be viewed as an adjunct for symptom and hospitalization control [15]. As the heart failure landscape continued evolving with sacubitril/valsartan (PARADIGM-HF, 2014) and ivabradine (US Food and Drug Administration (FDA)-approved 2015; incorporated into the 2016 ACC/AHA/Heart Failure Society of America (HFSA) focused update), digoxin held onto its class IIa status [16]. The 2022 AHA/ACC/HFSA guideline marked a second downgrade, from class IIa to class IIb (“might be considered in symptomatic HFrEF despite GDMT”), reflecting the uncertain benefit of digoxin in the context of contemporary four-pillar therapy [17]. This shift coincided with the rise of sodium-glucose cotransporter 2 (SGLT2) inhibitors as the fourth pillar of heart failure management, a status formalized by both the European Society of Cardiology (ESC) and ACC/AHA guidelines following the DAPA-HF and EMPEROR-Reduced trials [17, 18].

Another factor in digoxin’s decline was accumulating observational data. Meta-analyses of observational studies including *post hoc* analyses of trials such as AFFIRM reported that digoxin use was associated with a 15–38% increased risk of all-cause mortality in atrial fibrillation populations. However, these analyses were limited by confounding by indication, inconsistent SDC monitoring, and use of higher-than-optimal doses. Notably, a propensity-matched reanalysis of the same AFFIRM data found no difference in mortality [19].

## Digoxin in the Era of Quadruple Therapy: DIGIT-HF and DECISION

The benefit was evident, but the trial’s limitations also raised a critical and lasting question: does digoxin retain its benefit alongside modern four-pillar guideline-directed medical therapy (GDMT)? At the time of the DIG trial, background therapy was limited to ACE inhibitors and diuretics.

Three decades after the DIG trial, DIGIT-HF (2025) set out to re-examine the role of cardiac glycosides in heart failure, this time with digitoxin rather than digoxin. This international, double-blind, placebo-controlled trial enrolled 1,212 patients (modified intention-to-treat population; 1,240 randomized) with advanced heart failure with reduced ejection fraction (HFrEF) (LVEF ≤ 40% with New York Heart Association (NYHA) class III–IV, or LVEF ≤ 30% with NYHA class II) already on contemporary GDMT [20]. Digitoxin was started at 0.07 mg daily, with a median follow-up of 36 months. The primary composite endpoint (all-cause death or first hospitalization for worsening heart failure) was significantly reduced in the digitoxin group: 39.5% vs. 44.1% with placebo (HR, 0.82; 95% CI, 0.69–0.98; P = 0.03) [20]. Looking at the components individually, first hospitalization for worsening heart failure occurred in 28.1% vs. 30.4% (HR, 0.85; 95% CI, 0.69–1.05), and all-cause mortality in 27.2% vs. 29.5% (HR, 0.86; 95% CI, 0.69–1.07) neither reaching statistical significance on its own [20]. The treatment effect appeared consistent across prespecified subgroups. The trial used digitoxin rather than digoxin specifically to exploit its hepatic metabolism and longer half-life, which confer pharmacokinetic stability independent of renal function [20]. In this sense, DIGIT-HF addressed a question that had gone unresolved for nearly three decades: whether cardiac glycosides still offer benefit on top of contemporary four-pillar GDMT.

Shortly after DIGIT-HF, the DECISION trial (2026) examined low-dose digoxin (target SDC 0.5–0.9 ng/mL) in 1,001 patients with HFrEF or heart failure with moderately reduced ejection fraction (HFmrEF, LVEF ≤ 50%) on contemporary GDMT [8]. In DECISION, dosing was standardized to low maintenance regimens (0.0625 mg to 0.125 mg daily) adjusted for age, renal function, and body mass to consistently maintain low serum drug concentrations [8, 21]. The mean age was 72 years, 28% were women, and 29% had atrial fibrillation, with a median follow-up of 36.5 months [21]. The primary composite endpoint —total worsening heart failure events (hospitalizations and urgent visits) plus cardiovascular mortality—showed a rate ratio of 0.81 (95% CI, 0.61–1.07; P = 0.133), which did not reach statistical significance [21]. Total worsening heart failure events were numerically reduced (rate ratio 0.76; 95% CI, 0.54–1.05), while cardiovascular mortality was neutral (HR, 0.93; 95% CI, 0.69–1.26) [8]. Low-dose digoxin was well tolerated and safe, with consistent results between men and women. [21] Rather than representing a true biological contradiction, the DIGIT-HF and DECISION results are better understood as concordant in direction but divergent in statistical significance. DIGIT-HF met its primary endpoint (HR, 0.82; 95% CI, 0.69–0.98), and DECISION produced a nearly identical point estimate (rate ratio 0.81; 95% CI, 0.61–1.07) that did not reach significance, with a consistent reduction in worsening heart failure events (rate ratio 0.76) [21]. The most likely explanation lies in population risk and endpoint design. DIGIT-HF enrolled an advanced cohort (70% NYHA III–IV, mean LVEF about 29%) and used a time-to-first-event composite, whereas DECISION enrolled a less symptomatic population (88% NYHA class I or II, LVEF ≤ 50%) and employed a recurrent-events composite requiring an N-terminal pro-B-type natriuretic peptide (NT-proBNP) entry threshold; the lower absolute event rate left DECISION underpowered, generating fewer primary events despite a similar treatment effect. This mirrors, in a limited sense, the MITRA-FR versus COAPT experience, in which patient selection and baseline risk governed whether a real treatment effect reached significance—though unlike that pair, the digoxin trials did not diverge in the direction of effect [22, 23]. Whether the pharmacologic differences between digitoxin (used in DIGIT-HF) and digoxin (DECISION) contributed is possible but unlikely to be the principal factor, as the pooled analysis by Damman et al detected significant heterogeneity by glycoside type [24]. DECISION enrolled a less advanced heart failure population than DIGIT-HF and was likely underpowered for its recurrent-events primary endpoint.

Together, these trials began to establish a real benefit for cardiac glycosides, both digoxin and digitoxin. Damman et al performed a study-level meta-analysis pooling DIG, DIGIT-HF, and DECISION, spanning 9,013 patients and approximately 30 years of heart failure therapy [24]. The primary composite of cardiovascular death or first worsening heart failure event was significantly reduced with digitalis glycosides (HR, 0.85; 95% CI 0.80–0.90; P < 0.001) [12]. This benefit was driven by a reduction in worsening heart failure events (HR, 0.75; 95% CI, 0.69–0.81; P < 0.001), with no significant effect on cardiovascular mortality (HR, 0.99; 95% CI, 0.92–1.07) or all-cause mortality (HR, 0.97; 95% CI, 0.90–1.04) alone. There was no significant heterogeneity between the 1997 DIG trial and the contemporary trials, despite vastly different background therapies. While Damman et al suggested that cardiac glycosides demonstrate a consistent event-reduction effect across eras, describing digoxin as a potential “fifth pillar” requires substantial caution; its incremental therapeutic benefit remains modest compared to foundational quadruple therapies, particularly in patients already receiving maximally optimized GDMT [24]. The authors placed this effect size in context: the roughly 15% relative risk reduction with digitalis glycosides is smaller than that seen with SGLT2 inhibitors, similar to ivabradine, and larger than the pooled effect of vericiguat.

## A New Frontier: Digoxin in RHD (DIG-RHD)

While these studies debated digoxin’s place in conventional heart failure populations, the Dig-RHD trial addressed an entirely different and arguably more consequential clinical question: its use in RHD. This question carries deep weight for RHD patients, a population with a global prevalence of approximately 40.5 million (GBD 2019) and an estimated mortality burden of approximately 306,000 deaths annually (GBD 2019), though earlier estimates placed this figure at roughly 320,000 (GBD 2015) [25, 26]. These numbers matter not only for their scale but because the population affected is concentrated in low- and middle-income countries [5]. Despite this enormous burden, modern heart failure therapies had never been specifically tested in RHD.

Dig-RHD randomized 1,769 patients with symptomatic RHD across 12 centers in India to receive digoxin (0.125–0.25 mg daily) or placebo, with a median follow-up of approximately 2.1 years [5]. Specifically, in the digoxin group (885 patients), study drug was given at 0.125–0.25 mg once daily on a 5-days-per-week schedule (i.e., with 2 digoxin-free days per week). The most common regimen was 0.25 mg once daily for 5 days/week (about 50%), followed by 0.125 mg once daily for 5 days/week (about 40%), with the remainder on other regimens [5]. Dosing was individualized according to standard determinants (age, body weight, and renal function); patients with kidney insufficiency were excluded. The mean age was 46 years, and 72% of participants were female. Approximately 70% had atrial fibrillation or atrial flutter, and 90% were in NYHA class II–IV. Most patients (81.5%) had mixed lesions involving the mitral and aortic valves, and 85% had moderate to severe mitral stenosis (average mitral valve area 1.2 cm^2^). The mean LVEF was 58% [5].

The primary composite of all-cause death or new-onset/worsening heart failure was significantly reduced: 31.4% with digoxin vs. 35.5% with placebo (HR, 0.82; 95% CI, 0.70–0.97; P = 0.02) [5]. New-onset or worsening heart failure alone showed an HR of 0.82 (95% CI, 0.66–0.98), while all-cause mortality showed an HR of 0.94 (95% CI, 0.70–1.26). The number needed to treat was approximately 14 over 2 years to prevent one composite event. Pre-specified subgroup analyses showed greater benefit in atrial fibrillation (HR, 0.75; 95% CI, 0.62–0.90) than in sinus rhythm (HR, 1.08; P for interaction = 0.052), and in patients already on digoxin at baseline (HR, 0.61; 95% CI, 0.46–0.81; P for interaction = 0.01). The benefit was also more pronounced in women (HR, 0.78; 95% CI, 0.65–0.95) than in men (HR, 0.93; 95% CI, 0.69–1.26), and in patients with body mass index (BMI) < 18.5 (HR, 0.60; 95% CI, 0.40–0.92) compared with BMI ≥ 18.5 (HR, 0.88; 95% CI, 0.74–1.05) [5]. However, these secondary exploratory findings are viewed as hypothesis-generating rather than definitive evidence of selective efficacy.

Taken together, the Dig-RHD trial establishes digoxin as the first evidence-based medical therapy specifically tested for heart failure in RHD, a condition affecting more than 40 million people worldwide, disproportionately in low- and middle-income countries [20, 22]. Given this burden, and given how affordable and widely available digoxin is, the drug appears uniquely suited to this setting. Whether these trials will prompt a guideline upgrade from class IIb back toward class IIa, or the creation of RHD-specific recommendations, remains to be seen in forthcoming guideline updates.

The summary of all the trials related to digoxin are provided in Table 1.

**Table 1:** Summary of the Major Trials Related to Digoxin

Characteristic	DIG (1997)	DIGIT-HF (2025)	DECISION (2026)	DIG-RHD (2026)
Journal	NEJM	NEJM	Nature Medicine	JAMA
Drug	Digoxin	Digitoxin	Digoxin (low-dose)	Digoxin
Sample size (n)	6,800	1,212 (mITT)	1,001	1,769
Population	HFrEF (EF ≤ 45%), sinus rhythm	HFrEF (EF ≤ 40% NYHA III–IV, or ≤ 30% NYHA II)	HFrEF/HFmrEF (EF ≤ 50%)	Symptomatic RHD
Age (mean)	65 years	About 65 years	72 years	46 years
Sex (% female)	22%	About 22%	28%	72%
Atrial fibrillation	0% (excluded)	About 25%	29%	70%
Background therapy	ACEi (about 95%), diuretics (about 80%); no BB, MRA, ARNI, or SGLT2i	BB, ACEi/ARB/ARNI, MRA, SGLT2i (contemporary GDMT)	BB (86%), ACEi/ARB/ARNI (89%), MRA (72%), SGLT2i (41%)	β-blockers, CCBs, diuretics, anticoagulants (usual care for RHD)
Follow-up (median)	37 months	36 months	36.5 months	About 24 months
Primary endpoint	All-cause mortality	Death or first HF hospitalization	CV death or total worsening HF events	Death or new-onset/worsening HF
Primary endpoint result	RR 0.99	HR 0.82 (0.69–0.98); P = 0.03	RR 0.81 (0.61–1.07); P = 0.133	HR 0.82 (0.70–0.97); P = 0.02
Mortality outcome	No effect (RR 0.99)	HR 0.86 (0.69–1.07)	HR 0.93 (0.69–1.26)	HR 0.94 (0.70–1.26)
HF hospitalization/worsening HF	↓ 28% (P < 0.001)	HR 0.85 (0.69–1.05)	RR 0.76 (0.54–1.05)	HR 0.82 (0.69–0.98); P = 0.03

ACEi: angiotensin-converting enzyme inhibitor; ARB: angiotensin II receptor blocker; ARNI: angiotensin receptor-neprilysin inhibitor; BB: beta blocker; CCB: calcium channel blocker; CV: cardiovascular; EF: ejection fraction; GDMT: guideline-directed medical therapy; HF: heart failure; HFmrEF: heart failure with moderately reduced ejection fraction; HFrEF: heart failure with reduced ejection fraction; HR: hazard ratio; mITT: modified intention-to-treat; MRA: mineralocorticoid receptor antagonist; NYHA: New York Heart Association; RHD: rheumatic heart disease; RR: relative risk; SGLT2i: sodium-glucose cotransporter 2 inhibitor.

## Limitations and Considerations

Several limitations warrant consideration. First, the number of patients evaluated across contemporary trials remains modest relative to the trial programs supporting foundational GDMT, and the benefit is largely confined to a reduction in worsening heart failure events without a survival advantage; a pooled analysis confirmed benefit driven by heart failure events (HR = 0.75) with neutral cardiovascular and all-cause mortality [24].

Second, digitoxin (DIGIT-HF) and digoxin (DECISION, Dig-RHD) differ pharmacologically. Digitoxin has a far longer half-life and undergoes hepatic metabolism with renal-independent clearance, so findings for one cannot be extrapolated to the other [20].

Third, Dig-RHD was conducted only in India, in a predominantly female, relatively young cohort with excellent baseline renal function, as patients with kidney insufficiency were excluded; the investigators cautioned that the favorable safety profile may not extend to older patients with vascular or renal disease, limiting generalizability to North American or European populations [5].

Fourth, background therapy intensity varied across trials, and digoxin’s incremental efficacy may plausibly be smaller under maximal four-pillar GDMT. This remains theoretical, however: neither the DIGIT-HF subgroup analysis nor the Damman et al era-stratified pooled analysis detected significant effect modification by background therapy [24].

Finally, digoxin’s narrow therapeutic-to-toxic ratio—potentiated by hypokalemia, hypomagnesemia, impaired renal function, and interacting drugs—demands vigilant monitoring of renal function, electrolytes, and drug levels, infrastructure that cannot be assumed in the low- and middle-income settings where RHD is concentrated [7].

## Future Directions and Conclusions

Dig-RHD was conducted entirely at centers in India, in a population that was predominantly female, relatively young, and heavily weighted toward atrial fibrillation and mixed mitral-aortic disease, so confirmatory trials in other geographic and valvular populations would help establish how broadly the findings generalize, particularly to patients in sinus rhythm, where the subgroup interaction suggests the benefit may be attenuated.

The more immediate question is how guideline committees might weigh the evidence now in hand. Digoxin currently holds a class IIb recommendation in the 2022 AHA/ACC/HFSA guideline, reflecting uncertainty about its benefit atop four-pillar GDMT. The pooled DIG, DIGIT-HF, and DECISION data, which suggest a reduction in worsening heart failure events across three decades of changing background therapy, may prompt reconsideration of that position, though this evidence does not speak to RHD, a population no guideline has previously addressed with a tested medical therapy. Whether future updates move toward a class IIa recommendation in HFrEF and HFmrEF, introduce RHD-specific recommendations, or both, is a question that forthcoming revisions will need to weigh against a body of randomized evidence that has evolved since the last guideline cycle.

There may also be a meaningful implementation case. Digoxin is inexpensive, orally administered, and already included on essential medicines lists in the countries where RHD is concentrated, which sets it apart from newer agents that dominate contemporary heart failure guidelines but remain largely inaccessible in much of the world. A number needed to treat of approximately 14 over two years, applied to a condition affecting more than 40 million people, could translate into a meaningful public health effect at low marginal cost, though formal cost-effectiveness analyses in low- and middle-income settings would be needed to assess this more rigorously. Realizing any such benefit would also depend on the monitoring infrastructure a narrow-therapeutic-index drug requires, including access to serum levels, electrolytes, and renal function testing, which cannot be assumed in many of the settings where the drug would be used.

Taken together, the current evidence does not support digoxin as a mortality-reducing therapy, but it does suggest a potentially reproducible, low-cost option for reducing heart failure events —now supported by randomized data in a population that has not previously had a tested medical therapy available to it.

## Data Availability

The authors declare that data supporting the findings of this study are available within the article.
